# Title: Serious COVID-19 may have a causal relationship with myocardial injury: A Mendelian randomization study

**DOI:** 10.3389/fgene.2023.1135887

**Published:** 2023-03-23

**Authors:** Mei Jia Liu, Xue Qing Sun, Long Bo Li, Guan Wang, Yong Feng Shi

**Affiliations:** ^1^ Department of Ultrasound, The Second Hospital, Jilin University, Changchun, China; ^2^ Department of Cardiology, The Second Hospital, Jilin University, Changchun, China

**Keywords:** myocardial injury, SARS-CoV-2, COVID-19, Mendelian randomization, troponin I

## Abstract

**Background:** The association of coronavirus disease 2019 (COVID-19) with myocardial injury is not well known. This study explored the association between them using the Mendelian randomization (MR) method.

**Method:** We obtained summary data from genome-wide association studies (GWAS) on myocardial injury and COVID-19 from public databases. Then, as tool variables, we chose single nucleotide polymorphisms associated with susceptibility and COVID-19 severity to investigate the causal relationship of COVID-19 with myocardial injury using inverse-variance weighting (IVW) as the primary approach. Finally, the reliability of the results was evaluated by performing sensitivity analyses.

**Results:** As revealed by the IVW analyses, the seriously hospitalized patients with COVID-19 had causality with myocardial injury, with an β of 0.14 and 95% confidence interval (CI) of 0.03–0.25 (*p* = 0.01). The results showed that COVID-19 with severe respiratory symptoms positively affected myocardial injury (*β* = 0.11, 95% CI = 0.03–0.19; *p* = 0.005).

**Conclusion:** According to this study, severe respiratory symptoms and hospitalization due to COVID-19 may increase the risk of myocardial injury.

## Introduction

Coronavirus disease 2019 (COVID-19), a pandemic caused by severe acute respiratory syndrome coronavirus-2 (SARS-CoV-2), remains a global threat, seriously disrupting the economy and healthcare system. According to a World Health Organization report, there have been 50,400,000 confirmed COVID-19 cases and 6,200,000 deaths from COVID-19 as of April 20, 2022 (World Health Organization, 2022). New insights into how to treat and comprehend COVID-19 are desperately needed.

During their hospital stay, approximately 34% of COVID-19 patients experience cardiac damage, as evidenced by increased troponin levels in blood analysis (Zhao et al., 2020). When compared to its ancestors, SARS-CoV-2 appears to have more conclusive observational evidence identifying it as a cause of cardiac injury. Researchers discovered a significant correlation between severe disease, critical illness, and myocardial damage and inflammation indicators such as increased N-terminal pro-b-type natriuretic peptide, cardiac troponin I (cTnI), and high sensitivity C-reactive protein in a cross-sectional study (Chen et al., 2020). One of the early trials conducted in Wuhan showed that troponin I (high sensitivity cTnI) rises in 5 out of 41 patients with COVID-19 (Huang et al., 2020). Multiple mechanisms through which COVID-19 affects the cardiovascular system lead to myocardial damage and dysfunction and an increase in morbidity in individuals with underlying cardiovascular diseases. Unbalanced oxygen supply and demand, the creation of a prothrombotic environment, and endothelial dysfunction are the leading causes of cardiac damage in COVID-19 (Pericas et al., 2020).

Because of the defects of conventional statistical methods, such as confounding factors and bias, the stated associations between COVID-19 and cardiac injury could not be accurately determined. It is unclear how COVID-19 and myocardial injury are causally associated.

Mendelian randomization (MR) strategy can overcome the above constraints (Smith and Ebrahim, 2003; Smith and Ebrahim, 2004). MR study explores causality by using genes as instrumental variables (IVs), and the distribution of genes in the population follows the second law of Mendel (Smith and Ebrahim, 2003). Moreover, it is a randomized controlled study based on Mendelian law. In MR studies, exposure-outcome causality was explored using single nucleotide polymorphisms (SNPs) as the IVs of exposure. If a direction, such as COVID-19, contributes to an event, such as cardiac injury, a variable that affects COVID-19 should also be expected to contribute to cardiac injury. MR analysis was used in this study to investigate the relationship between COVID-19 and cardiac injury.

## Material and methods

**FIGURE 1:**
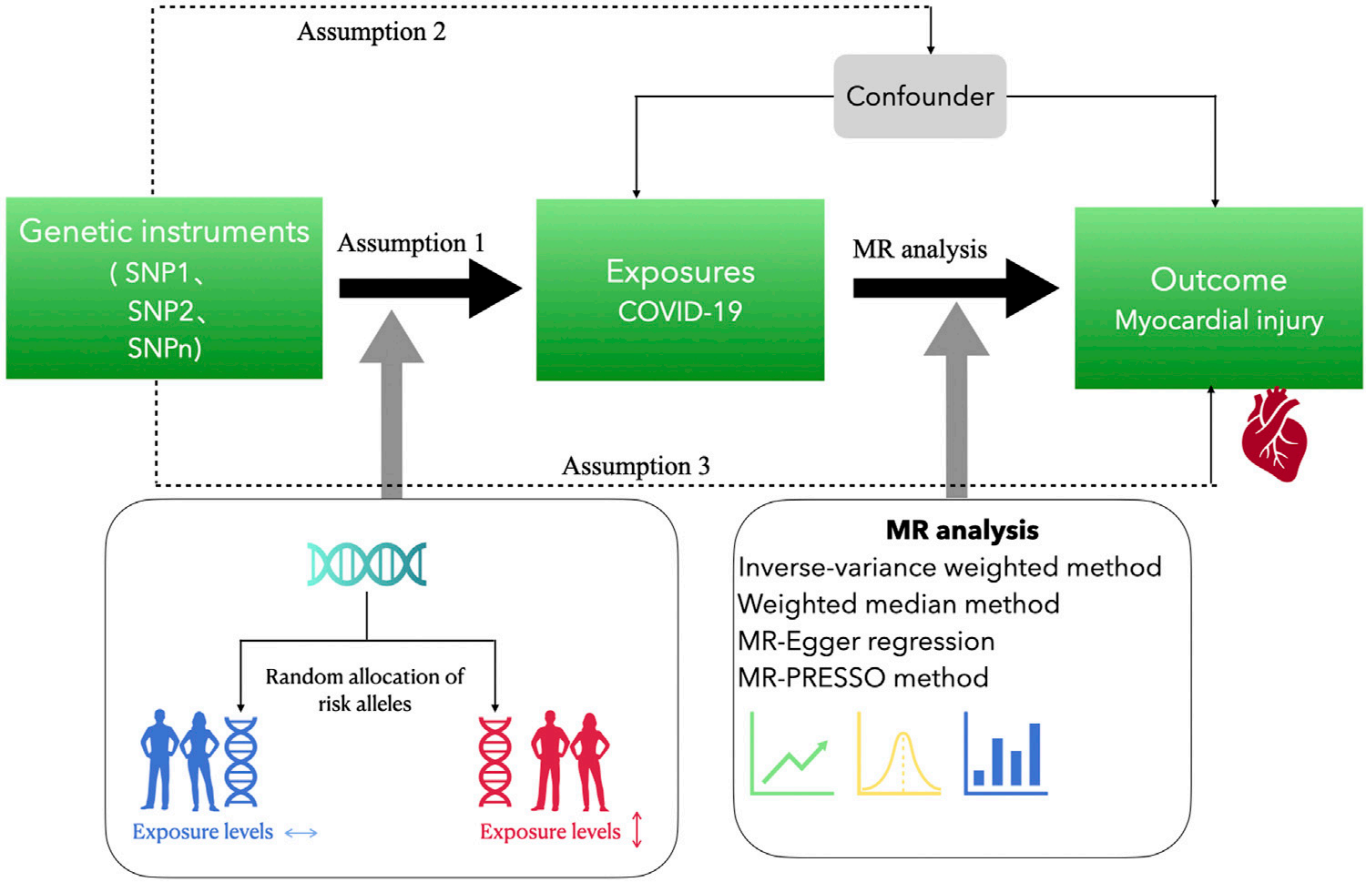
Study design of the Mendelian randomization study. Assumption 1 is that the genetic instrumental variables are associated with exposure to COVID-19 and assumption 2 is that the genetic instrumental variables are not associated with the outcome *via* a confounding pathway. Assumption 3 is that the genetic instrumental variables do not affect the outcome directly, only possibly indirectly *via* the exposure. Assumption 1 is tested by statistical methods, such as *p*-value <5 × 10^−8^, F statistic >10, inter-SNP linkage disequilibrium r^2^ > 0.001, and clump distance = 10,000 kb. Assumption 2 is tested by using PhenoScanner. Assumption 3 is tested by genetic instrumental variables *p* > 5 × 10^−8^.

Different GWAS data sources were explored to collect the genetic tools for exposure and outcome using a two-sample MR design (Figure 1). The COVID-19-cardiac injury causality was assessed using two-sample MR analyses, where some genetic variants from the publicly accessible GWAS summary data were used. This was a re-analysis of information that had already been acquired and published. No further ethical approval was necessary.

### Data source

The exposure data of COVID-19 phenotypic statistics in this study were sourced from the latest GWAS meta-analyses by the COVID-19 Host Genetics Initiative (Round 7, April 2022) (The COVID-19 Host Genetics Initiative, 2020), including GWAS summary data concerning various COVID-19 outcomes. Among the 2,942,817 European ancestry participants recruited in the seventh release, 159,840 were COVID-19 cases (control cases = 2,782,977), 44,986 were COVID-19-induced hospitalization cases (control cases = 2,356,386), and 18,152 developed very severe respiratory symptoms as a result of COVID-19 (control cases = 1,145,546).

The outcome variable data for cardiac injury (troponin I) were obtained from a GWAS analysis study that included 2,994 plasma proteins from 3,301 European descendants (Sun et al., 2018). There were no individuals who overlapped between the exposure and outcome studies.

### Instrumental variable selection

The genetic variants were selected as IVs to calculate the causative effects of COVID-19 on cardiac injury: Being a predictor of COVID-19, being free of confounding factors, and no association with TNI except *via* COVID-19. From the relevant genome-wide association studies, genetic tools that met a *p* < 5 × 10^−8^ genome-wide significance criterion were selected. In our analysis, if the Mendelian randomization results showed inconsistency, we used more stringent criteria to select IVs (Ong and MacGregor, 2019). Inter-SNP linkage disequilibrium (LD; r^2^ < 0.001, clump distance = 10,000 kb) was evaluated using the 1000 Genomes European reference panel (https://www.internationalgenome.org). When suitable proxies were available, missing SNPs from an outcome dataset were substituted (minimum LD, r^2^ = 0.8). We eliminated all palindromic SNPs and SNPs without an imputed replacement.

We used the exposure and eliminated them to prevent reverse causality in MR analysis. Any possible outliers were recognized and removed through the MR-Pleiotropy Residual Sum and Outlier (MR-PRESSO) test to reduce the effect of outliers.

### Statistical analyses

The primary analytical approach adopted in this study was the inverse-variance weighting (IVW), (Bowden et al., 2015) using which the causality between COVID-19 and troponin I was examined. When *p* ≤ 0.05, the relationship was considered statistically significant. We used MR-Egger and weighted median procedures (wider confidence intervals [Cis]) to supplement IVW computations, which, despite lower efficiency, could yield more accurate estimates in a wider range of scenarios.

### Sensitivity analyses

In MR studies, sensitivity analysis is critical for the underlying pleiotropy recognition, whereas heterogeneity is perhaps seriously violated for MR estimations. The heterogeneity markers (Cochran Q-derived *p* < 0.05) from the IVW technique were used to demonstrate potential horizontal pleiotropy. MR-Egger regression intercept was used to recognize the directional pleiotropy (defined as the existence of *p* < 0.05) (Burgess and Thompson, 2017). Meanwhile, horizontal pleiotropy was assessed and corrected using MR-PRESSO (Ong and MacGregor, 2019), which included 1) detecting horizontal pleiotropy, 2) correcting it through outlier elimination, and 3) testing for statistically significant differences between causal estimates before and after outlier rectification. Compared with IVW and MR-Egger, it is less biased and more accurate at fluctuation rates of horizontal pleiotropy below 10%.

The effect of a single SNP on the MR calculation was also assessed using the leave-one-out method. The possible directional pleiotropy was evaluated using a funnel plot, a tool also used in meta-analyses to identify publication bias.

Analyses were performed with the aid of R (ver. 4.2.0) (R Core Team, 2021) packages, TwoSampleMR (ver. 0.5.6) (Hemani et al., 2018) combined with MRPRESSO (ver. 1.0) (Verbanck, 2017).

## Results

### The results of instrumental variable selection

Apart from weak IVs (F statistic < 10), the entire significant (*p*-value >5 × 10^−8^, stricter *p*-value >1 × 10^−9^) and independent (r^2^ > 0.001) SNPs about COVID-19 vulnerability and severity were eliminated from the two-sample MR analyses. A total of 11 vulnerability-associated SNPs (mean of F statistic = 47.09), 29 COVID-19 hospitalization because of COVID-19-associated SNPs (mean of F statistic = 30.98), and 20 severe respiratory symptoms arising from COVID-19-associated SNPs (mean of F statistic = 34.51) were retained for MR analysis.

### Causal effect of COVID-19 vulnerability on cardiac injury

**FIGURE 2:**
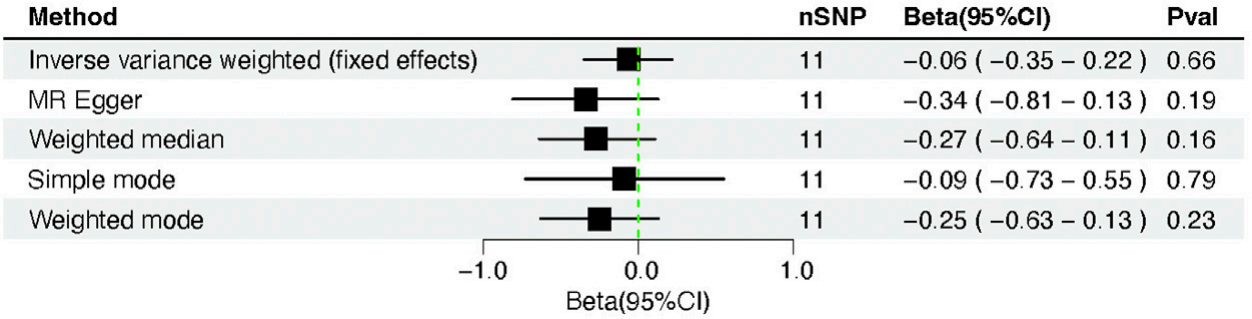
Forest plot of Mendelian randomization analysis between COVID-19 susceptibility and cardiac injury risk.

The causal connection of COVID-19 vulnerability with myocardial injury was assessed by using MR-Egger, IVW (fixed effects), weighted median, and simple and weighted modes (Figures 2, 5). Since the results of the simple mode method were inconsistent with those of other methods, we changed the *p*-value threshold to 1 × 10^−9,^ and 11 SNPs were used as instrumental variables. When we chose the criterion of *p* < 5 × 10^−8^ for the selection of instrumental variables, the results of the MR analysis showed that the Simple mode method yielded a β value of 0.437, whereas the other methods yielded β values of, respectively, MR Egger of −0.281, Weighted median is −0.214, IVW is −0.022, and Weighted mode is −0.234. The results showed that the β values obtained for Simple mode and the other methods were not in the same direction, so we adjusted the criteria for the instrumental variables to make the selection of instrumental variables more stringent. We tried to gradually reduce the *p*-values from 5 × 10^−8^ to 1 × 10^−8^, 5 × 10^−9^, or even 1 × 10^−9^. When we lowered it to 1 × 10^−8^, the β value obtained by the simple mode method was 0.187, still not in the same direction as the β values obtained by other methods, until we lowered the *p*-value to 1 × 10^−9^, the β value of Simple mode agreed with the β values measured by other methods, with a β value of −0.067, the in the same direction as the β values obtained by the other methods.

We did not discover evidence of a possible relationship between COVID-19 susceptibility and cardiac injury based on the IVW (β = −0.06, 95% CI = −0.35–0.22; *p* = 0.66). However, neither the MR-Egger regression (β = −0.34, 95% CI = −0.81–0.13; *p* = 0.19) nor the weighted median technique (β= −0.27, 95% CI = −0.64–0.11; *p* = 0.16) yielded statistically significant results. Moreover, the above result was confirmed by the simple mode method (β= −0.09, 95% CI = −0.73–0.55; *p* = 0.79) and the weighted mode approach (β= −0.25, 95% CI = −0.63–0.13; *p* = 0.23) (Table 1).

**TABLE 1:** Mendelian randomization outcomes between the vulnerability of COVID-19 and cardiac injury.

Method	nSNP	Beta	Se	Pval	Beta(95%CI)
IVW	11	−0.06	0.14	0.66	−0.06 (−0.35–0.22)
MR Egger	11	−0.34	0.24	0.19	−0.34 (−0.81–0.13)
Weighted median	11	−0.27	0.18	0.14	−0.27 (−0.62–0.08)
Simple mode	11	−0.09	0.33	0.79	−0.09 (−0.73–0.55)
Weighted mode	11	−0.25	0.22	0.28	−0.25 (−0.67–0.18)

The Cochran Q-test revealed that the *p*-value for MR-Egger was 0.47 and for IVW was 0.38, indicating no heterogeneity. No directional pleiotropy was observed (intercept = 0.02; standard error [SE] = 0.01; *p* = 0.19). In addition, MR-PRESSO reported a similar *p*-value of 0.40 for the globe heterogeneity evaluation. The MR-PRESSO test revealed the absence of significant outliers.

In summary, the COVID-19 vulnerability was not causally linked to the cardiac injury biomarkers troponin I. According to the leave-one-out sensitivity assessments, the overall effect of COVID-19 susceptibility and troponin I was not violated significantly by any SNP (Figure 6).

### Causal effect of severity-COVID-19-induced hospitalization on cardiac injury

**FIGURE 3:**
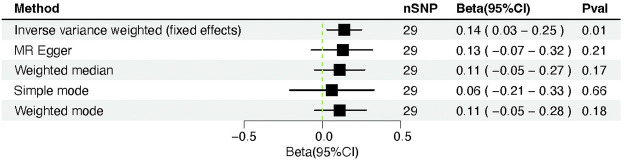
Forest plot of Mendelian randomization analysis between COVID-19 severity (hospitalization) and cardiac injury risk.

The severity-COVID-19-induced hospitalization was linked causally to the cardiac injury, supported by the IVW’s (IVW fixed effects) findings (β = 0.14, 95% CI = 0.03–0.25; *p* = 0.01) (Table 2). Other MR approaches, including MR-Egger (β = 0.13, 95% CI = −0.07–0.32; *p* = 0.21), weighted median (β = 0.11, 95% CI = −0.05–0.27; *p* = 0.18), simple mode (β = 0.06, 95% CI = −0.20–0.33; *p* = 0.65), and weighted mode (β = 0.11, 95% CI = −0.06–0.29; *p* = 0.20) also produced results that were consistent but not statistically significant (Figures 3, 5).

**TABLE 2:** Mendelian randomization analysis between COVID-19 severity (hospitalization) and cardiac injury risk.

Method	nSNP	Beta	Se	Pval	Beta(95%CI)
IVW	29	0.14	0.06	0.01	0.14 (0.03–0.25)
MR Egger	29	0.13	0.10	0.21	0.13 (−0.07–0.32)
Weighted median	29	0.11	0.08	0.17	0.11 (−0.05–0.27)
Simple mode	29	0.06	0.14	0.66	0.06 (−0.21–0.33)
Weighted mode	29	0.11	0.08	0.18	0.11 (−0.05–0.28)

The Cochran Q-test yielded *p*-values of 0.90 for MR-Egger and 0.92 for IVW, indicating that there was no heterogeneity. Furthermore, MR-PRESSO achieved a similar result (the *p*-value in the global heterogeneity test was 0.93). Furthermore, no outliers were discovered using the MR-PRESSO method. No significant intercept could be demonstrated (intercept = −0.002; SE = 0.009; *p* = 0.85), suggesting no directional pleiotropy was found. The leave-one-out assessment suggested the stability of the foregoing results (Figure 6).

We used PhenoScanner (http://www.phenoscanner.medschl.cam.ac.uk) to check the correlation of COVID-19 hospitalization instrument variables with other phenotypes. The results showed that some SNPs (“rs2068205”, “rs1634761”, “rs646327”) in instrument variables were associated with rheumatoid arthritis, high cholesterol, hypertension, and cardiovascular disease. We removed these SNPs and then performed Mendelian randomization analysis again and found that it did not affect the causal relationship between COVID-19 and myocardial injury (IVW: β = 0.14, 95% CI = 0.03–0.26; *p* = 0.01).

### Causal effect of severity-COVID-19-induced severe respiratory symptoms on cardiac injury

The results of the MR analysis showed that the β value obtained by the Simple mode method was −0.043, while the β values obtained by the other methods were, respectively, 0.030 for MR Egger, 0.075 for Weighted median, −0.022 for IVW and −0.234 for Weighted mode. The results showed that the β value obtained by the Simple mode method was not in the same direction as those obtained by the other methods, so we adopted the criterion of adjusting the instrumental variables so that the β results obtained by the various methods were in the same direction, and adopted a more stringent criterion of selecting the instrumental variables. We tried to gradually reduce the *p*-value from 5 × 10^−8^ gradually lowered to 1 × 10^−8^, 5 × 10^−9^, or even 1 × 10^−9^. When we lowered it to 1 × 10^−8^, the β value obtained by the Simple mode method was 0.185, the β value of Simple mode was consistent with the β values measured by other methods, so we did not continue to lower the instrumental variable *p*-value criterion.

**FIGURE 4:**
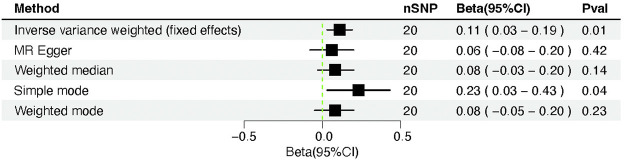
Forest plot of Mendelian randomization analysis between COVID-19 severity (severe respiratory symptoms) and cardiac injury risk.

**FIGURE 5:**
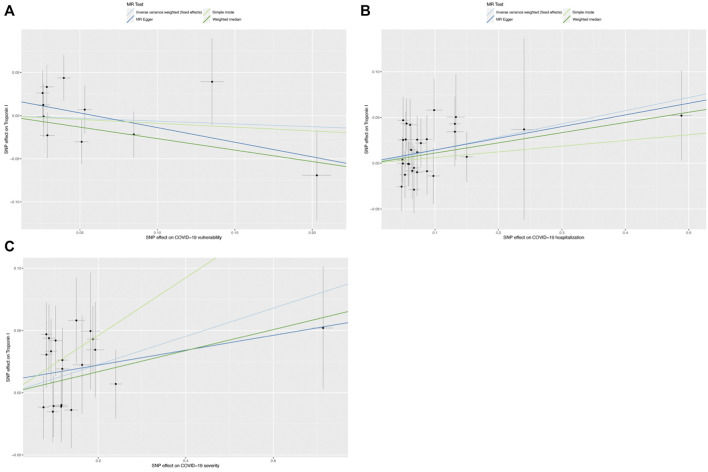
Scatter graphs for single-nucleotide polymorphisms (SNPs) concerning COVID-19 and cardiac injury. **(A)** Scatter graph for SNPs concerning COVID-19 vulnearability and cardiac injury risk; **(B)** Scatter graph for SNPs concerning COVID-19 severity (hospitalization) and cardiac injury risk, and **(C)** Scatter plot of SNPs concerning the severity of COVID-19 (severe respiratory symptoms) and risk of cardiac injury.

We changed the p threshold value to 1 × 10^−8^ and introduced 20 SNPs as instrumental variables because the simple mode method results were inconsistent with other methods’ results. The IVW fixed effects method identified a significant causality between severity-COVID-19-induced severe respiratory symptoms and cardiac injury (β = 0.11, 95% CI = 0.03–0.19; *p* = 0.005) (Figures 4, 5). Identical risk estimates (β = 0.08, 95% CI = −0.03–0.20; *p* = 0.14) were derived with the weighted median method, despite the statistically insignificant association. Consistent results were obtained using the MR-Egger approach (β = 0.06, 95% CI = −0.08–0.20; *p* = 0.42), albeit with a statistically insignificant association. Moreover, the result derived using the Simple mode (β = 0.23, 95% CI = 0.02–0.44; *p* = 0.04) was significant, and the result obtained by the weighted mode method (β = 0.08, 95% CI = −0.03–0.19; *p* = 0.19) resembled those with the weighted median and MR-Egger techniques (Table 3).

**TABLE 3:** Shows the Mendelian randomization analysis between COVID-19 severity (severe respiratory symptoms) and cardiac injury risk.

Method	nSNP	Beta	Se	Pval	Beta(95%CI)
IVW	20	0.11	0.04	0.01	0.11 (0.03–0.19)
MR Egger	20	0.06	0.07	0.42	0.06 (−0.08–0.20)
Weighted median	20	0.08	0.06	0.14	0.08 (−0.03–0.20)
Simple mode	20	0.23	0.10	0.04	0.23 (0.03–0.43)
Weighted mode	20	0.08	0.06	0.23	0.08 (−0.05–0.20)

**FIGURE 6:**
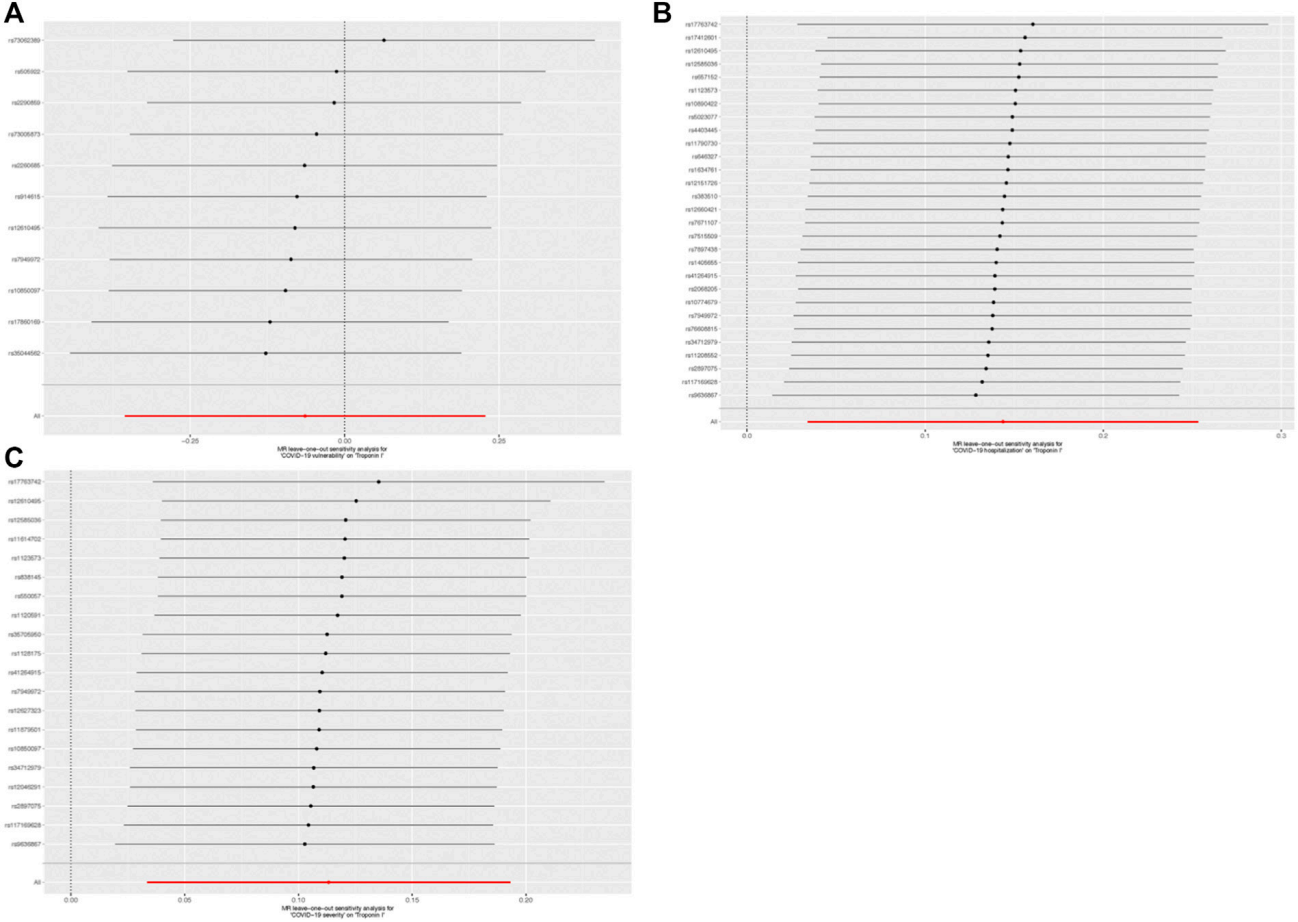
Leave-one-out outcome for single-nucleotide polymorphisms (SNPs) concerning COVID-19 and cardiac injury. **(A)** Leave-one-out outcome for SNPs concerning COVID-19 vulnerability and cardiac injury risk; **(B)** Leave-one-out outcome for SNPs concerning COVID-19 severity (hospitalization) and cardiac injury risk; and **(C)** Leave-one-out outcome for SNPs concerning the severity of COVID-19 (severe respiratory symptoms) and cardiac injury risk.

No heterogeneity was observed using a Cochran Q-test, which showed *p*-values of 0.71 and 0.72 for MR-Egger and IVW, respectively. Meanwhile, MR-PRESSO revealed a similar *p*-value of 0.74 in examining the global heterogeneity. Furthermore, no outliers were discovered. Furthermore, the intercept value was 0.01 (SE = 0.01; *p* = 0.39), indicating that no directional pleiotropy was observed. No single SNP strongly violated the overall effect during the leave-one-out sensitivity test (Figure 6).

We used PhenoScanner (http://www.phenoscanner.medschl.cam.ac.uk) to check the correlation of severity-COVID-19-induced severe respiratory symptoms instrument variables with other phenotypes. The results showed that some SNPs (“rs550057", “rs1128175", “rs838145”) in instrument variables were associated with rheumatoid arthritis, alcohol intake, hypertension, diabetes, and cardiovascular disease. We removed these SNPs and then performed Mendelian randomization analysis again and found that it did not affect the causal relationship between COVID-19 and myocardial injury (IVW: β = 0.12, 95% CI = 0.04–0.21; *p* = 0.003).

### Bidirectional Mendelian randomization study to rule out reverse causality

The causal relationship between COVID-19 and myocardial injury was studied using bidirectional Mendelian randomization. Based on the IVW, we found no evidence of a causal relationship between cardiac injury and COVID-19 susceptibility (OR = 1.01, 95% CI = 0.98–1.03; *p* = 0.70). And cardiac injury was not associated with COVID-19 hospitalization (OR = 1.03, 95% CI = 0.97–1.09; *p* = 0.31) and COVID-19-induced severe respiratory symptoms (OR = 1.06, 95% CI = 0.98–1.15; *p* = 0.17).

## Discussion

This study examined whether genetically predicted COVID-19 determinants cause an increase in cardiac injury using a two-sample MR method. Three large GWASs selected candidate IVs for COVID-19 susceptibility and severity-hospitalization and severity-severe respiratory symptoms. The IVs had a higher chance of correctly predicting COVID-19 (F-statistics > 10). Our findings indicated that a severe COVID-19 infection could exacerbate heart damage. Patients with COVID-19, particularly those in a severe state, should be given much more attention to prevent cardiac injury.

According to previous COVID-19 research, SARS-CoV-2 directly binds to angiotensin-converting enzyme 2 (ACE2) receptors, resulting in cellular entry (South et al., 2020). The ACE2 receptor is highly expressed in the lung, the heart, the kidneys, and other organs. Therefore, in addition to the clinical respiratory manifestations such as respiratory failure and severe pneumonia, increasing evidence suggests that the cardiovascular system is also affected by COVID-19, mainly by cardiac injury. Cardiac damage was noted in 16.8% of 2895 patients with COVID-19 in a study, defined by troponin levels above normal (Smilowitz et al., 2021). In another study in Italy, cardiac injury (cardiac troponin 1 elevation) was presented in 27.2% of 313 patients with COVID-19 (De Michieli et al., 2021). A total of 288 out of 434 (66.4%) patients with COVID-19 suffered from a cardiac injury, according to a multicenter trial conducted in the United Kingdom with higher percentages (Papageorgiou et al., 2022). Among several patients with COVID-19, the rates of cardiac injury are higher (Metkus et al., 2021; Kozlik et al., 2022).

Aside from myocardial ischemia, non-ischemic myocardial disorders such as myocarditis, pulmonary embolism, sepsis, acute respiratory infections with hypoxia, and cardiac adrenergic hyperstimulation in cytokine storm syndrome can all cause cardiac injury (Imazio et al., 2020). Evidence indicates a link between high troponin levels and a bad outcome in COVID-19 (Guo et al., 2020; Shi et al., 2020).

The causes are believed to be multifaceted and include direct consequences such as myocarditis and indirect types of harm such as demand ischemia or thrombosis caused by the underlying respiratory problem. The hearts of several patients with COVID-19 had microvascular coronary thrombi during autopsies (Basso et al., 2020). COVID-19-associated micro- and macroangiopathic thromboembolic complications may lead to coronary microvascular thrombosis and cause cardiac injury with increased troponin I levels (Tersalvi et al., 2020). According to an autopsy study (Bois et al., 2021), cardiac fibrin microthrombi are frequently found in patients with COVID-19. No concrete proof of a direct myocardial infarction exists. And myocarditis was discovered in 33.3% of patients.

In recent studies, cardiomyocytes from autopsies and endomyocardial biopsies have been found to contain SARS-CoV-2 particles or components (Gauchotte et al., 2021). An underlying factor in the COVID-19 heart condition could be direct SARS-CoV-2 damage to the cardiomyocytes.

In recent studies, cardiomyocytes from autopsies and endomyocardial biopsies have been found to contain SARS-CoV-2 particles or components (Gauchotte et al., 2021). Direct SARS-CoV-2 damage to cardiomyocytes could be an underlying factor in the COVID-19 heart condition.

This study uses the two-sample MR methodology to understand previous observational data better. The observational study on the relationship between COVID-19 and cardiac damage mentioned above cannot establish a causal relationship due to bias. Earlier studies made various adjustments for these risk factors, and confounding risk factor violations were challenging to eliminate from earlier observational studies.

This study shows that the myocardial risk can be increased by severe COVID-19. Therefore, it is necessary to routinely screen the markers of myocardial damage for patients with COVID-19, particularly those in a severe state. For patients with myocardial injury, various treatments should be taken to reduce myocardial injury.

This study provides several benefits. First, because of the MR approach, this study perhaps initially resembled an observational randomized controlled study. However, expensive and frequently challenging randomized controlled trials are widely used to examine causes. When SNPs are given randomly, confounding bias can be evaded by MR investigations. In addition, MR can manage the reverse causal influence, unlike earlier observational studies. Second, on the basis of our findings, patients with severe COVID-19 need to receive greater attention to reduce cardiac injury.

However, there were certain limitations. First, the GWAS data were sourced entirely from most European individuals, and it needs further validation if these findings can be generalized. The causal relationship between COVID-19 and myocardial injury in other ethnic groups needs to be investigated further. COVID-19 data for East Asian, Hispanic, South Asian, and African populations are currently available. The causal relationship between COVID-19 and myocardial injury in different populations can be studied further in the future with larger sample sizes. Second, although MR studies exploited the random distribution of the genetic variations to establish causal hypotheses, it was not easy to completely distinguish mediation and pleiotropy. One or multiple phenotypes may be affected by significant differences in our genome. Third, no mediator analysis was performed in this study.

## Conclusion

Our MR findings support the hypothesis that COVID-19 may exacerbate the cardiac injury.

## Data Availability

The original contributions presented in the study are included in the article/Supplementary Material, further inquiries can be directed to the corresponding author.

## Abbreviations

COVID-19, Coronavirus disease 2019; SARS-CoV-2, Severe acute respiratory syndrome coronavirus-2; MR, Mendelian randomization; GWAS, Genome-wide association studies; IVW, Inverse-variance weighting; OR, Odds ratio; CI, Confidence interval; cTnI, Cardiac troponin I; IVs, Instrumental variables; SNPs, Single nucleotide polymorphisms; LD, Linkage disequilibrium; MR-PRESSO, MR-Pleiotropy Residual Sum and Outlier methods; SE, Standard error; ACE2, Angiotensin-converting enzyme 2.
